# Supplementary material for: Chemical modification of hyaluronan oligosaccharides differentially modulates hyaluronan–hyaladherin interactions
Source: J Biol Chem. 2024 Aug 14;300(9):107668. doi: 10.1016/j.jbc.2024.107668 (PMC11460632; doi:10.1016/j.jbc.2024.107668)
Supplement: Supplemental Tables S1-S11 and Figure S1-S11 [file mmc1.pdf]

Chemical modification of hyaluronan oligosaccharides differentially modulates hyaluronan-hyaladherin interactions.

Rebecca J. Dodd[1,2], Charles D. Blundell[3], Benedict M. Sattelle[3], Jan J. Enghild[4], Caroline M. Milner[1,2,5] and Anthony J. Day[1,2,5*]

[1]Wellcome Trust Centre for Cell Matrix Research, School of Biological Sciences, Faculty of Biology, Medicine & Health, University of Manchester, Manchester, M13 9PL

[2]Lydia Becker Institute of Immunology and Inflammation, University of Manchester, Manchester, M13 9PT, United Kingdom

[3]C4X Discovery, 53 Portland Street, M1 3LD, Manchester, United Kingdom

[4]Department of Molecular Biology and Genetics, Aarhus University, Aarhus, Denmark

[5]Division of Cell Matrix Biology and Regenerative Medicine, School of Biological Sciences, Faculty of Biology, Medicine & Health, University of Manchester, Manchester, M13 9PL, United Kingdom.

*Corresponding author: Professor Anthony J. Day; email: anthony.day@manchester.ac.uk; telephone: +44 (0)161 2751495.

Supporting Information Contents **Page**

A. Characterization of modified HA oligosaccharides.

Table S1	Amine-containing reagents used to chemically modify HA_n^{AN} oligosaccharides.	S-3
Table S2	Molecular masses for unmodified and modified HA oligosaccharides.	S-3
Figure S1	Example MALDI-TOF mass spectra of modified HA.	S-4

B. Investigation of the interaction of HA-2AA modified oligosaccharides with Link_TSG6 by NMR spectroscopy.

Figure S2	[^{1}H-^{15}N]-HSQC spectra of Link_TSG6 in the presence of unmodified and modified HA hexasaccharides.	S-5
Figure S3	[^{1}H-^{15}N]-HSQC spectra of Link_TSG6 in the presence of unmodified and modified HA octasaccharides.	S-5
Figure S4	Modified HAn-AA oligosaccharides are accommodated in the HA-binding groove of Link_TSG6	S-6

C. Purification and characterization of the new CD44_HABD construct.

Figure S5	Purification and characterization of hisCD44_HABD$^{20\text{-}169}$.	S-7

D. Determination of the solution dynamic 3D structure of HA_4-2AA.

Table S3	^{1}H chemical shifts for HA_4-2AA.	S-8
Table S4	^{13}C chemical shifts for HA_4-2AA.	S-9
Table S5	Temperature coefficients (^{1}H $\Delta\delta_{HN}/\Delta T$) of exchangeable amide and aniline hydrogen nuclei in HA_4-2AA compared to HA_4^{AN}.	S-10
Table S6	Conformation-dependent scalar coupling constants for residue T4 of HA_4-2AA (modified GlcNAc) compared to β-configuration GlcNAc rings in HA_4^{AN}.	S-10

Table S7	Conformational restraints derived from scalar couplings and their χ^2 fits.	S-10
Table S8	NOE conformational restraints for HA_4-2AA and their χ^2 fits.	S-11
Table S9	noNOE conformational restraints for residues G3-X5 in HA_4-2AA and their χ^2 fits.	S-12
Table S10	Summary of χ^2 fits for all structural restraints for residues G3-X5 in HA_4-2AA.	S-13
Table S11	Torsion angle conformational preferences for HA_4-2AA in aqueous solution.	S-14
Figure S6	2D structure and nomenclature of HA_4-2AA.	S-15
Figure S7	pH titration of HA_4-2AA.	S-15
Figure S8	Chemical shift perturbations in HA_4-2AA compared to HA_4^{AN}.	S-16
Figure S9	Solution dynamic 3D structure of HA_4-2AA in a conformational ensemble representation	S-17
Figure S10	Comparison of solution conformational behavior of HA_4-2AA with HA_5^{AA}.	S-18

E. Generation of a model of the HA_6-2AA/Link_TSG6 complex.

Figure S11	Comparison of conformations of HA_8^{AN} bound to Link_TSG6 with the modeled conformations of HA_6-2AA used for docking into the binding site of Link_TSG6.	S-19

F. Supporting Information References. S-20

A. Characterization of modified HA oligosaccharides.

Table S1. Amine-containing reagents used to chemically modify HA_n^{AN} oligosaccharides.

Chemical	Abbreviated name	Modified HA oligosaccharide
2-aminobenzoic acid	2AA	HA_n•2AA
3-aminobenzoic acid	3AA	HA_n•3AA
4-aminobenzoic acid	4AA	HA_n•3AA
2-amino-4-methoxy benzoic acid	2A4MBA	HA_n•2A4MBA
3-amino-pyrazole	3APY	HA_n•3APY
4-aminoindan	4AI	HA_n•4AI
5-aminoindan	5AI	HA_n•5AI
6-amino-quinolone	6AQ	HA_n•6AQ

Table S2. Molecular masses for unmodified and modified HA oligosaccharides.

HA	Formula	$[M-H]^-$ (Da) [a]	Observed mass (Da) [b]	Difference (Da) [c]
**HA_4^{AN}**	$C_{28}H_{44}N_2O_{23}$	775.6	775.7 ±0.1	0.1
**HA_4-2AA**	$C_{35}H_{49}N_3O_{24}$	895.6	894.3 ± 0.1	1.3
**HA_4-3AA**	$C_{35}H_{49}N_3O_{24}$	895.6	896.5 ± 0.2	0.9
**HA_4-4AA**	$C_{35}H_{49}N_3O_{24}$	895.6	896.9 ± 0.7	1.3
**HA_4-2A4MBA**	$C_{36}H_{52}N_3O_{25}$	927.3	926.1 ± 0.3	1.2
**HA_6^{AN}**	$C_{42}H_{65}N_3O_{34}$	1154.9	1155.1 ±0.2	0.2
**HA_6-2AA**	$C_{49}H_{70}N_4O_{35}$	1275.5	1275.6 ± 0.1	0.1
**HA_6-3AA**	$C_{49}H_{70}N_4O_{35}$	1275.5	1275.3 ± 0.1	0.2
**HA_6-4AA**	$C_{49}H_{70}N_4O_{35}$	1275.5	1274.8 ± 0.2	0.7
**HA_6-2A4MBA**	$C_{50}H_{73}N_4O_{36}$	1306.6	1305.5 ± 0.7	1.1
**HA_6-3APY**	$C_{45}H_{69}N_6O_{33}$	1221.1	1221.5 ± 0.1	0.4
**HA_6-4AI**	$C_{51}H_{75}N_4O_{33}$	1271.1	1271.5 ± 0.1	0.4
**HA_6-5AI**	$C_{51}H_{75}N_4O_{33}$	1271.1	1271.7 ± 0.1	0.6
**HA_6-6AQ**	$C_{51}H_{72}N_5O_{33}$	1282.1	1282.3 ± 0.0	0.2
**HA_8^{AN}**	$C_{56}H_{86}N_4O_{45}$	1534.3	1534.5 ±0.2	0.2
**HA_8-2AA**	$C_{63}H_{91}N_5O_{46}$	1654.7	1654.9 ± 0.1	0.2
**HA_8-3AA**	$C_{63}H_{91}N_5O_{46}$	1654.7	1654.6 ± 0.6	0.1
**HA_8-4AA**	$C_{63}H_{91}N_5O_{48}$	1654.7	1654.9 ± 0.1	0.2
**HA_8-2A4MBA**	$C_{64}H_{94}N_5O_{47}$	1686.0	1687.3 ± 0.4	1.3
**HA_8-3APY**	$C_{59}H_{90}N_7O_{44}$	1600.4	1600.4 ± 0.1	0.0
**HA_8-4AI**	$C_{65}H_{96}N_5O_{44}$	1650.5	1651.1 ± 0.4	0.6
**HA_8-5AI**	$C_{65}H_{96}N_5O_{44}$	1650.5	1651.1 ± 0.1	0.6
**HA_8-6AQ**	$C_{65}H_{94}N_6O_{44}$	1661.5	1661.7 ± 0.5	0.2

[a] Theoretical value of $[M-H]^-$ based on the average molecular mass of oligosaccharide. [b] Determined from negative-ion MALDI-TOF mass spectra; value is the average mass recorded on three individual sample spots ± standard deviation. [c] Difference between the theoretical mass and the observed mass.

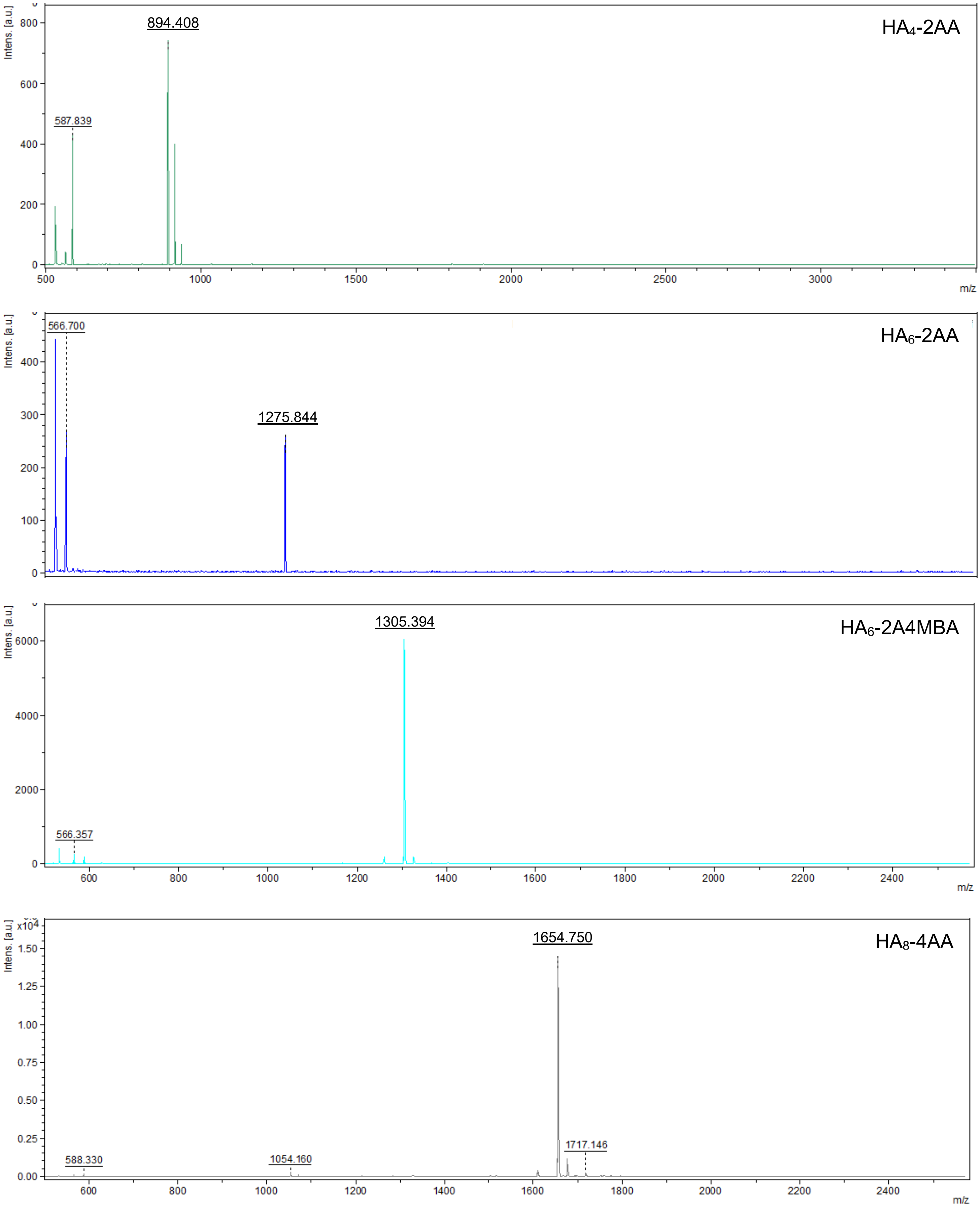

Figure S1. Example MALDI-TOF mass spectra of modified HA. Spectra are representative of those recorded on three individual samples. Additional species (500 Da) can be attributed to the MALDI analysis matrix (6-aza-2-thiothymine).

B. Investigation of the interaction of HA-2AA modified oligosaccharides with Link_TSG6 by NMR spectroscopy.

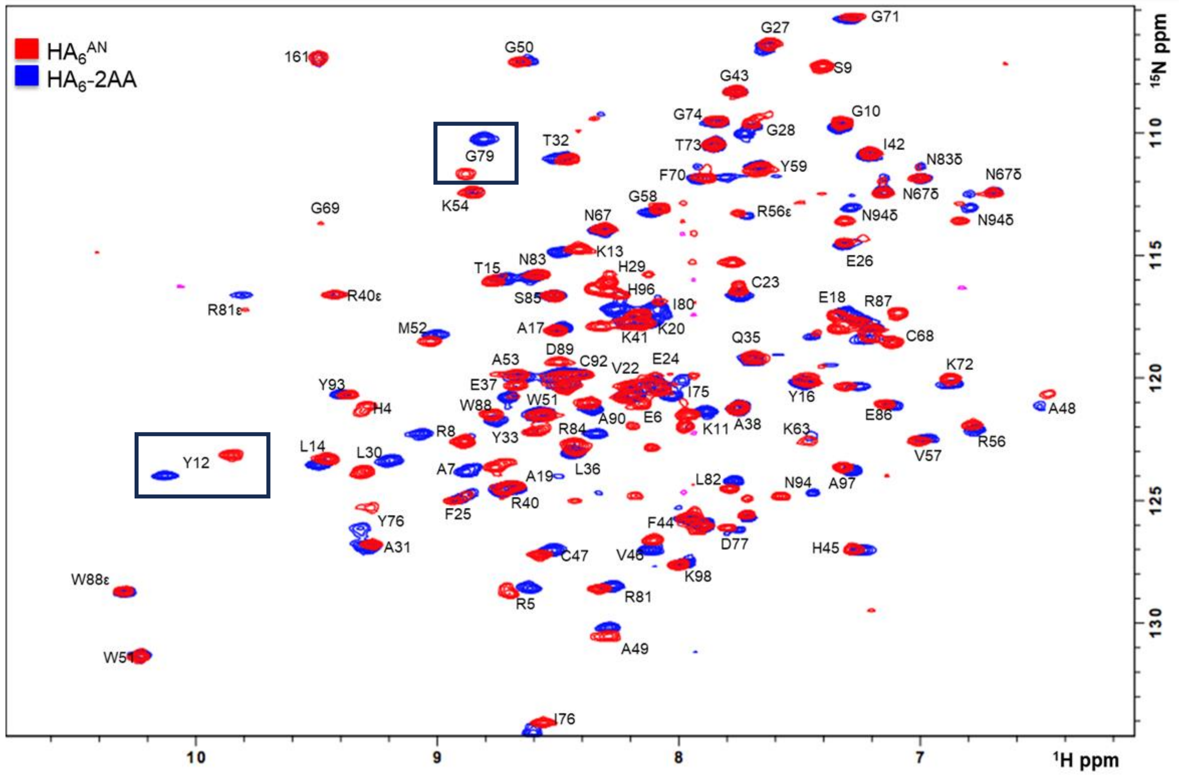

Figure S2. [^{1}H-^{15}N]-HSQC spectra of Link_TSG6 in the presence of unmodified and modified HA hexasaccharides. [^{1}H-^{15}N]-HSQC NMR spectra of 0.1 mM ^{15}N-Link_TSG6 in the presence of 10-fold molar excess of either HA_6^{AN} (red) or HA_6-2AA (blue). NH resonances are labelled in one letter code (backbone and sidechain) according to the assignment for Link_TSG6 reported previously (PDB accession code: 1o7c, (1)). Chemical shift perturbations of Tyr12 (Y12) and Gly79 (G79) are highlighted (square boxes).

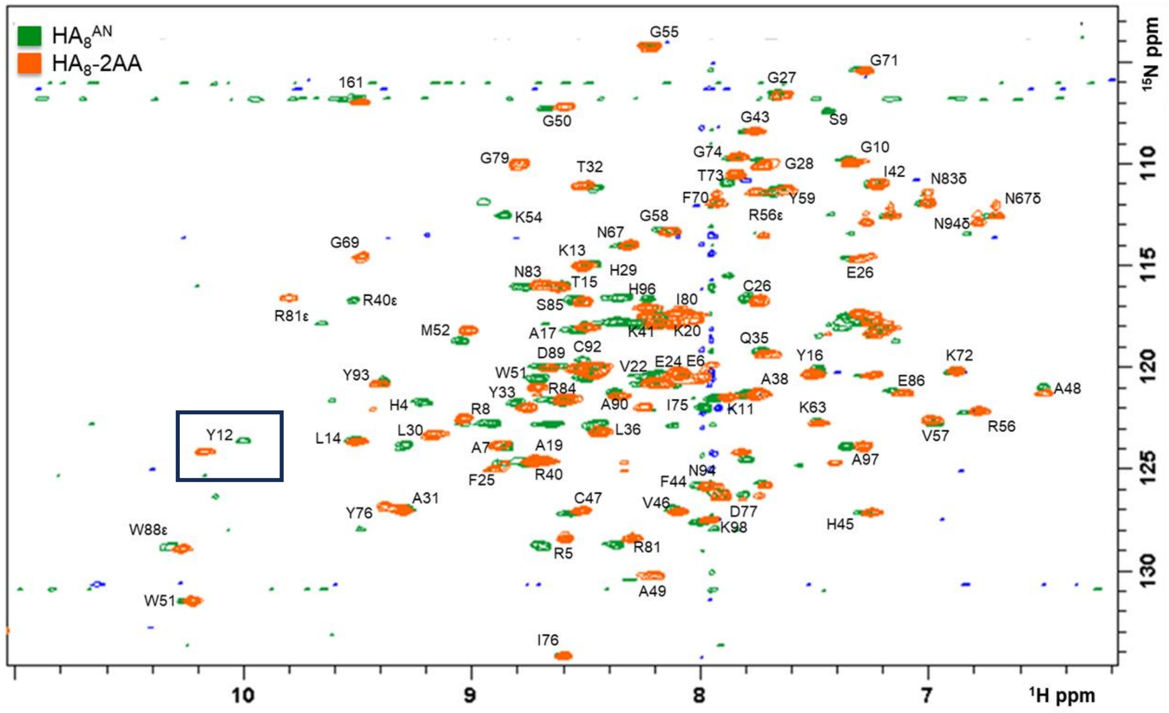

Figure S3. **[^{1}H-^{15}N]-HSQC spectra of Link_TSG6 in the presence of unmodified and modified HA octasaccharides.** [^{1}H-^{15}N]-HSQC NMR spectra of 0.1 mM ^{15}N-Link_TSG6 in the presence of 10-fold molar excess of either HA_8^{AN} (green) or HA_8-2AA (orange). NH resonances are labelled in one letter code (backbone and sidechain) according to the assignment for Link_TSG6 reported previously (pdb accession code: 1o7c (1)). Chemical shift perturbation of Tyr12 (Y12) is highlighted (square box).

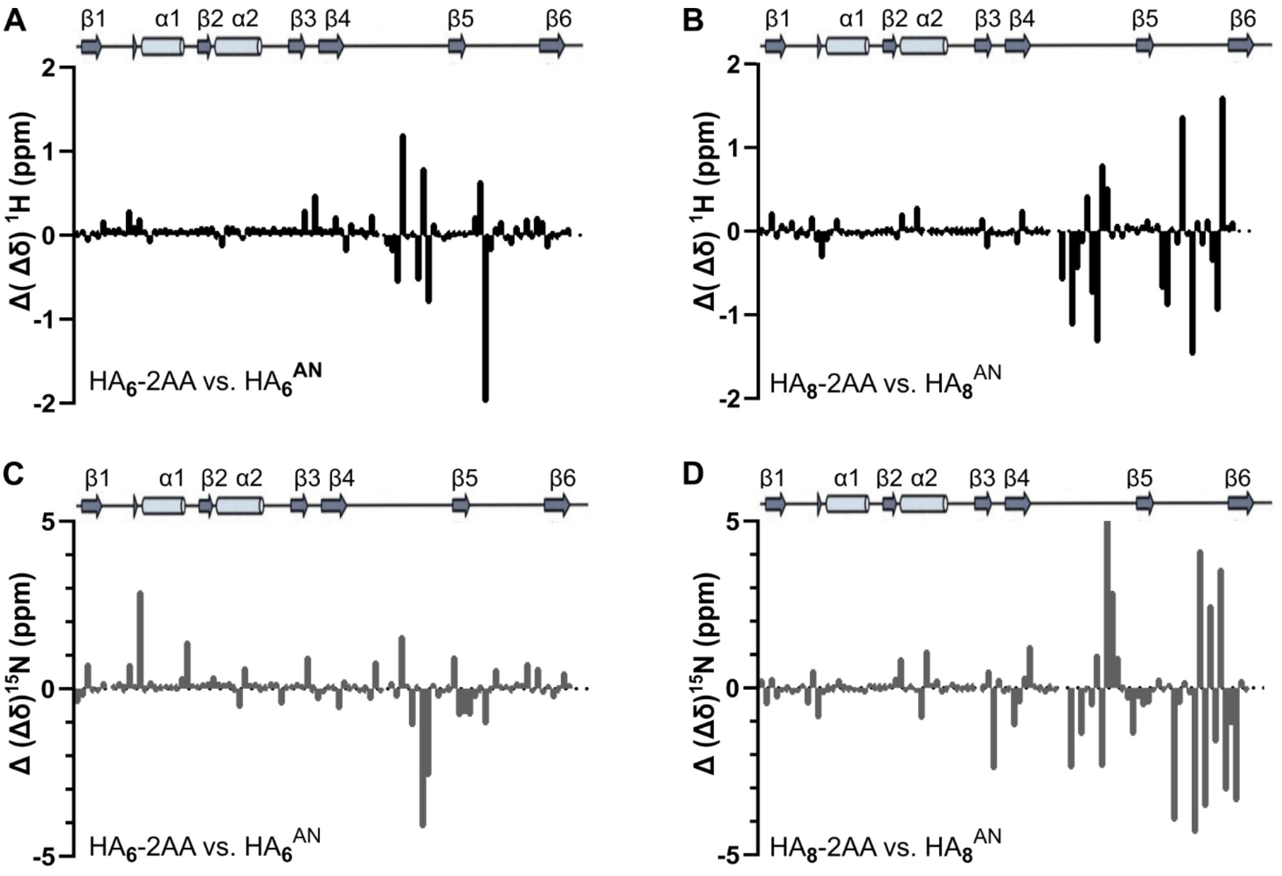

Figure S4. Modified HA_n-AA oligosaccharides are accommodated in the HA-binding groove of Link_TSG6. Chemical shift perturbations were measured from [^1H-^{15}N]-HSQC spectra recorded on ^{15}N-lablled Link_TSG6 in the presence of modified and unmodified HA oligosaccharides (see **Figure S2** and **Figure S3**). Plots show the change (Δ(Δδ)) in ^1H (**A**, **B**) and ^{15}N (**C**, **D**) chemical shifts for Link_TSG6 bound by HA_6-2AA compared to those induced by binding of HA_6^{AN} (**A**, **C**) and HA_8-2AA compared to HA_8^{AN} (**B**, **D**). In **A-D**, the secondary structure elements of Link_TSG6 (as determined in (41)) are shown above each graph.

C. Purification and characterisation the new construct for the HA-binding domain of CD44

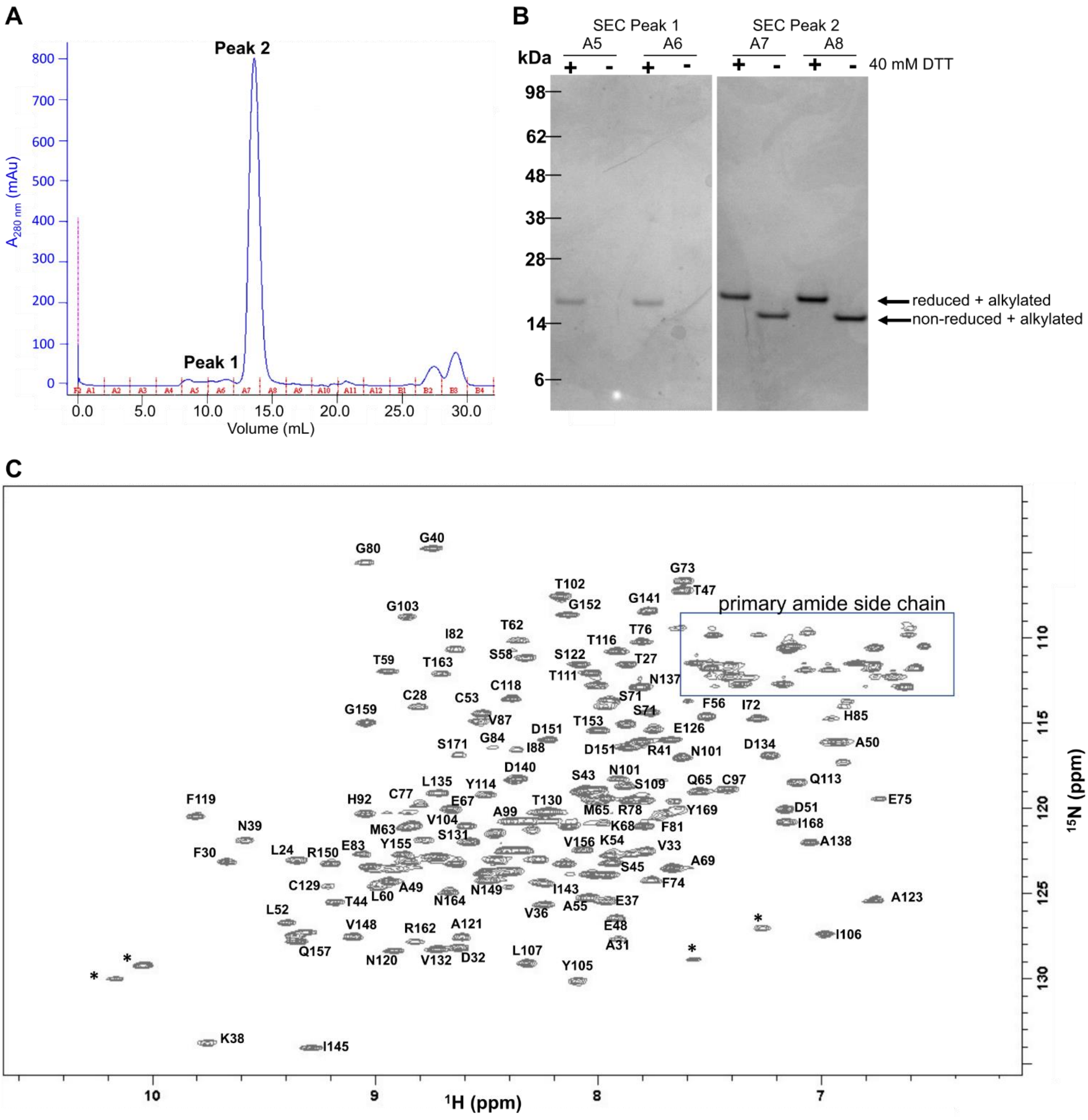

Figure S5. Purification and characterization of hisCD44_HABD$^{20\text{-}169}$. **A**) Size Exclusion Chromatography (SEC) of the hisCD44_HABD$^{20\text{-}169}$ construct following its expression *in E. coli*, refolding and initial purification on IMAC (not shown); the eluent was monitored continuously at A280$_{nm}$ and 2.5-mL fractions were collected. **B**) Fractions across Peak 1 (A5, A6) and Peak 2 (A7, A8) from SEC in (**A**) were analyzed under reduced (+ 40 mM DTT and alkylated) or non-reduced (alkylated) conditions. The majority of the hisCD44_HABD$^{20\text{-}169}$ protein was present in Peak 2, where this had a different apparent molecular weight on SDS-PAGE under reducing and non-reducing conditions (~20 and ~15 kDa, respectively), consistent with the presence of disulfide bonds; a band was only seen under reduced conditions for Peak 1 indicating this corresponds to aggregated material. **C**) 0.1 mM ^{15}N-hisCD44_HABD$^{20\text{-}169}$ was analyzed by [^{1}H-^{15}N]-HSQC NMR at pH 7.5 on a spectrometer operating at 500 MHz. The spectrum was essentially identical to that for CD44_HABD$^{20\text{-}178}$ (2), allowing for its assignment, except that it lacks the resonances from the absent 9 C-terminal residues, which considerably improves the resolution at the crowded center. The primary amide side chain region for Asn (N) and Qlu (Q) residues is indicated (box).***D. Determination of the solution dynamic 3D structure of HA$_4$-2AA.***

Table S3. ^{1}H chemical shifts for HA_4-2AA.

Residue	Atom	^{1}H δ (ppm) [a] HA_4^{AN} pH 6.0, 24.4 °C 10% (v/v) D_2O 5-20 mM	^{1}H δ (ppm) HA_4-2AA pH 9.4, 5.0 °C 10% (v/v) D_2O, 1 mM	^{1}H δ (ppm) HA_4-2AA pH 6.0, 5 °C 100% (v/v) D_2O, 3 mM	Δδ (ppm) [b] HA_4^{AN} pH 6.0 vs HA_4-2AA pH 9.4	Δδ (ppm) [c] HA_4-2AA pH 6.0 vs HA_4-2AA pH 9.4
**G1 GlcA**	H1 [d]	4.458 [e]	4.468	4.468	-0.010	0.000
	H2	3.321	3.323	3.317	-0.002	0.006
	H3	3.496	3.486	3.486	-0.010	0.000
	H4	3.497	3.485	3.485	0.012	0.000
	H5	3.723	3.725	3.725	-0.059	0.000
**S2 GlcNAc**	H1	4.560	4.561	4.561	-0.001	0.000
	H2	3.851	3.858	3.858	-0.007	0.000
	H3	3.707	3.705	3.705	0.002	0.000
	H4	3.542	3.554	3.554	-0.012	0.000
	H5	3.484	3.477	3.477	0.007	0.000
	H61	3.921	3.924	3.918	-0.003	0.006
	H62	3.780	3.792	3.792	-0.012	0.000
	HNA1	- [f]	8.202	8.198	-	0.004
	HA3*	2.028	2.034	2.024	-0.006	0.010
**G3 GlcA**	H1	4.467	4.578	4.578	-0.111	0.000
	H2	3.363	3.411	3.404	-0.048	0.007
	H3	3.580	3.620	3.620	-0.040	0.000
	H4	3.744	3.728	3.728	0.016	0.000
	H5	3.703	3.728	3.728	-0.025	0.000
**T4 GlcNAc**	H11	- [g]	3.646	3.646	-	0.000
	H12	- [g]	3.247	3.244	-	0.003
	H2	3.806	4.441	4.441	-0.635	0.000
	H3	3.716	4.065	4.062	-0.349	0.003
	H4	3.514	3.567	3.567	-0.053	0.000
	H5	3.472	3.846	3.846	-0.374	0.000
	H61	3.750	3.831	3.824	-0.081	0.007
	H62	3.890	3.606	3.606	0.284	0.000
	HNA1	- [f]	8.243	8.237	-	0.006
	HA3*	2.015	1.933	1.933	0.082	0.000
**X5 2AA**	HN11	- [g]	7.965	- [h]	-	-
	H2	- [g]	6.914	6.919	-	-0.005
	H3	- [g]	7.760	7.760	-	0.000
	H4	- [g]	6.771	6.776	-	-0.005
	H5	- [g]	7.374	7.374	-	0.000

[a] Chemical shifts for the β-anomer of HA_4^{AN} as reported in (3). [b] Chemical shift changes from HA_4^{AN} (as reported in (3)) including temperature and pH difference. [c] Refer to Figure S6 for nomenclature. [d] Chemical shift changes resulting from replacement of H_2O with D_2O and increase in pH. [e] All chemical shifts were referenced relative to internal DSS-d_6. Standard error on measurements: ^{1}H ± 0.001 ppm. [f] Resonance not visible due to exchange with D_2O. [g] Equivalent atom not present in HA_4^{AN}. [h] Resonance not visible at pH 6.0.

Table S4. ^{13}C chemical shifts for HA_4-2AA.

Residue	Atom	^{13}C δ (ppm) HA_4^{AN} [a]<br>pH 6.0, 24.4 °C<br>10% (v/v) D_2O<br>5-20 mM	^{13}C δ (ppm) HA_4-2AA<br>pH 9.4, 5.0 °C<br>10% (v/v) D_2O<br>1 mM	Δδ (ppm) [b]<br>HA_4^{AN} pH 6.0, 24.4 °C<br>vs.<br>HA_4-2AA pH 9.4, 5.0 °C
**G1 GlcA**	C1 [c]	105.715 [d]	105.707	0.008
	C2	75.578	75.364	0.214
	C3	78.213	77.971	0.242
	C4	74.560	74.443	0.117
	C5	78.477	78.614	-0.137
**S2 GlcNAc**	C1	103.325	103.827	-0.502
	C2	57.049	56.990	0.059
	C3	85.839	85.623	0.216
	C4	71.357	71.100	0.257
	C5	78.183	77.963	0.220
	C6	63.390	63.094	0.296
	CA3	25.326	25.212	0.114
**G3 GlcA**	C1	105.818	105.608	0.072
	C2	75.294	75.345	-0.051
	C3	76.506	76.159	0.347
	C4	82.781	82.551	0.230
	C5	79.098	78.586	-0.512
**T4 GlcNAc**	C1	97.879	46.087	51.502
	C2	58.454	53.001	5.453
	C3	85.151	79.482	5.669
	C4	71.351	73.363	-2.012
	C5	78.264	73.217	5.047
	C6	63.567	65.347	-1.780
	CA3	25.065	24.764	0.301
**X5 2AA**	C2	- [e]	115.966	-
	C3	- [e]	134.273	-
	C4	- [e]	119.769	-
	C5	- [e]	135.268	-

[a] Chemical shifts for the β-anomer of HA_4^{AN} as reported in (3). [b] Chemical shift changes from HA_4^{AN} including temperature and pH changes. [c] Refer to Figure S6 for nomenclature. [d] All chemical shifts were referenced indirectly relative to internal DSS-d_6. Standard error on measurements: ^{13}C ± 0.020 ppm. [e] Atom not present in HA_4^{AN}.

Table S5. Temperature coefficients ($\Delta\delta_H/\Delta T$) of exchangeable amide and aniline hydrogen nuclei in HA_4-2AA compared to HA_4^{AN}.

Residue	Atom	**HA_4-2AA** pH 6.0, 3 mM 100% (v/v) D_2O ppb/K	**HA_4-2AA** pH 9.4, 1 mM 10% (v/v) D_2O ppb/K	**HA_4^{AN}, pH 6.0** [a] pH 6.0, 5-20 mM 10% (v/v) D_2O ppb/K
**S2**	HNA1 [b]	-6.8 [c]	-6.9	-7.0
**T4**	HNA1	-6.8	-6.8	-[d]
**X5**	HN11	-[e]	-2.6	-[d]
H_2O [f]		-11.3	-11.3	-[d]

[a] Temperature coefficient as reported in (3). [b] Refer to Figure S6 for nomenclature. [c] Standard error on measurements: ± 0.1 ppb/K. [d] Equivalent atom not present in HA_4^{AN}. [e] HN was no longer observed due to fast chemical exchange with solvent at this pH value. [f] Temperature coefficient of water reported as internal control.

Table S6. Conformation-dependent scalar coupling constants for residue T4 of HA_4-2AA (modified GlcNAc) compared to β-configuration GlcNAc rings in HA_4^{AN}.

$^3J_{HH}$ coupling constant	HA_4-2AA [a] T4-GlcNAc residue (Hz)	HA_4^{AN} [b]		Mean Δ^3J_{HH} [c] (Hz)
		β-GlcNAc (Hz)	internal GlcNAc (Hz)	
H2-HNA1/HN [d]	9.8 [e]	9.70	9.68	0.1
H2-H3	6.1	-	10.37	4.3
H3-H4	1.7	8.81	-	7.2
H4-H5	8.5	9.73	10.00	1.4
H5-H61	-[f]	5.47	5.32	-
H5-H62	4.1	2.20	2.31	-1.7

[a] Values measured at pH 9.4, 5 °C, 1 mM in 10% (v/v) D_2O. [b] Values for β-GlcNAc and internal (I)-GlcNAc rings in HA_4^{AN} as reported in (3)).[c] Mean difference between values in residue T4 (of HA_4-2AA) compared to the same coupling constant in either the β-GlcNAc or internal GlcNAc ring of HA_4^{AN}. [d] Refer to Figure S6 for nomenclature. [e] All coupling constants shifts were determined by direct measurement from 1D spectra. Standard error on all couplings ± 0.05 Hz. [f] Value not measured due to spectral overlap and lineshape distortion caused by strong coupling.

Table S7. Conformational restraints derived from scalar couplings and their χ^2 fits.

**$^3J_{HH}$ coupling constant**	**Karplus parameters** [a]				**observed value (Hz)**	**calculation error applied (Hz)** [c]	**mean predicted value (Hz)**	**mean χ2**
	**A**	**B**	**C**	**phase Ψ**				
T4 H11-H2	8.804	-0.990	1.452	1	3.7 ± 0.05	± 0.75	3.22	0.497
T4 H12-H2	9.036	-0.983	1.336	-7	11.0 ± 0.05	± 0.75	1.05	0.489
T4HNA1-H2	9.450	-2.080	0.630	-5	9.8 ± 0.05	± 0.75	9.99	0.155
T4 H2-H3	7.564	-0.896	0.847	10	6.1 ± 0.05	± 0.75	5.58	0.068
T4 H3-H4[b]	7.516	-0.879	0.567	-15	1.7 ± 0.05	± 0.75	1.61	0.046
T4 H4-H5	6.578	-0.910	1.036	0	8.5 ± 0.05	± 0.75	8.35	0.099
T4 H62-H5	8.317	-0.990	1.371	-1	4.1 ± 0.05	± 0.75	4.17	0.123
X5 HN11-T4 H11	7.900	-1.050	0.650	-5	6.6 ± 0.05	± 0.75	6.25	0.369
X5 HN11-T4 H12	7.900	-1.050	0.650	-5	4.5 ± 0.05	± 0.75	4.59	0.415

[a] Karplus equation in the form: $^3J = A\cos^2(\theta+\Psi) + B\cos(\theta+\Psi) + C$, with Ψ in degrees (4). [b] Refer to Figure S6 for nomenclature. [c] Error applied to observed $^3J_{HH}$ value is that of predictive capability of the Karplus relation, rather than measurement error (5).

Table S8. NOE conformational restraints for HA_4-2AA and their χ^2 fits.

donor residue [a, b]	donor nuclei	acceptor residue	acceptor nuclei	measured height [c]	calculation error applied	mean predicted height	mean χ2
G3	H1	G3	H1	9100	3640	5690	0.885
G3	H3	G3	H1	1090	435	1120	0.018
G3	H4 & H5 [d]	G3	H1	2290	916	1940	0.151
T4	H12	G3	H1	293	117	108	2.510
T4	H2	G3	H1	365	146	224	0.940
T4	H3	G3	H1	1860	742	1520	0.209
T4	H4	G3	H1	131	52	181	0.979
G3	H4 & H5	G3	H2	1640	656	1380	0.151
T4	H2	G3	H2	54	22	48	0.104
T4	H3	G3	H2	138	57	85	0.864
T4	H5 & H61	G3	H2	201	80	143	0.747
T4	HA3*	G3	H2	28	12	25	0.194
G3	H1	G3	H3	1350	539	1120	0.196
G3	H3	G3	H3	10900	4360	11200	0.009
G3	H1	T4	H11	211	86	103	1.620
T4	H4	T4	H11	333	133	312	0.059
X5	H2	T4	H11	351	141	243	0.612
G3	H1	T4	H12	218	87	108	1.620
T4	H4	T4	H12	379	152	178	1.760
X5	H2	T4	H12	229	93	273	0.241
G3	H1	T4	H2	306	123	224	0.458
T4	H2	T4	H2	5820	2330	6650	0.167
T4	H4	T4	H2	370	148	443	0.261
T4	H5 & H61	T4	H2	123	49	98	0.268
X5	H2	T4	H2	339	136	308	0.138
G3	H1	T4	H3	2020	809	1520	0.387
G3	H2	T4	H3	89	36	85	0.019
G3	H4 & H5	T4	H3	495	198	361	0.457
T4	H5 & H61	T4	H3	430	172	326	0.384
T4	HA3	T4	H3	90	36	18	3.960
X5	H2	T4	H3	177	71	119	0.670
T4	H12	T4	H4	283	113	178	0.861
T4	H2	T4	H4	391	156	443	0.128
X5	H2	T4	H4	85	34	135	2.130
G3	H1	X5	H2	56	23	54	0.063
T4	H11	X5	H2	288	116	243	0.175
T4	H12	X5	H2	179	72	273	1.750
T4	H2	X5	H2	336	135	308	0.132
T4	H3	X5	H2	73	29	119	2.510
X5	H4	X5	H4	9780	3910	10500	0.041
X5	H5	X5	H5	7570	3040	10200	0.734

[a] The donor and acceptor nuclei are those on the indirect and direct dimensions of the spectrum, respectively. [b] Refer to Figure S6 for nomenclature. [c] Measured height, calculation error applied, predicted heights, and calculated mean χ^2, as described in (5)). Data measured from a 2D-NOESY recorded with 700 ms mixing time on a 3 mM sample at pH 6.0 in 100% (v/v) D_2O and at 5 °C. [d] These restraints were included as overlaps between the NOE cross-peaks indicated.

Table S9. noNOE conformational restraints for residues G3-X5 in HA_4-2AA and their χ^2 fits.

donor residue [a, b]	donor nuclei	acceptor residue	acceptor nuclei	measured height [c]	calculation error applied	mean predicted height	mean χ^2
X5	H3	G3	H1	0.0	33.3	1.6	0.002
X5	H4	G3	H1	0.0	32.3	2.1	0.004
X5	H5	G3	H1	0.0	41.1	9.6	0.055
T4	H12	G3	H2	0.0	55.5	23.9	0.192
X5	H2	G3	H2	0.0	18.0	9.7	0.294
X5	H3	G3	H2	0.0	14.3	4.1	0.088
X5	H4	G3	H2	0.0	19.4	1.7	0.008
X5	H5	G3	H2	0.0	13.2	2.9	0.050
T4	H12	G3	H3	0.0	29.4	19.8	0.463
T4	H2	G3	H3	0.0	44.8	27.1	0.365
T4	HA3	G3	H3	0.0	30.1	18.9	0.404
X5	H2	G3	H3	0.0	30.0	10.5	0.125
X5	H3	G3	H3	0.0	18.4	1.13	0.005
G3	H2	T4	H11	0.0	22.9	14.9	0.428
T4	H5	T4	H11	0.0	36.2	40.3	1.240
T4	H61	T4	H11	0.0	36.7	16.3	0.202
T4	HA3	T4	H11	0.0	17.5	5.1	0.085
X5	H3	T4	H11	0.0	13.3	6.1	0.212
X5	H4	T4	H11	0.0	14.3	15.1	1.130
G3	H2	T4	H12	0.0	39.0	23.9	0.389
T4	H5	T4	H12	0.0	30.0	25.2	0.705
T4	H61	T4	H12	0.0	26.8	8.62	0.106
T4	HA3	T4	H12	0.0	20.4	6.53	0.103
X5	H3	T4	H12	0.0	19.8	6.25	0.101
X5	H4	T4	H12	0.0	23.1	14.2	0.385
G3	H3	T4	H2	0.0	47.5	27.1	0.325
T4	H62	T4	H2	0.0	63.4	17.5	0.077
T4	HA3	T4	H2	0.0	46.9	37.9	0.652
X5	H3	T4	H2	0.0	22.7	6.1	0.071
X5	H4	T4	H2	0.0	21.7	8.9	0.169
X5	H3	T4	H3	0.0	22.4	2.4	0.011
X5	H4	T4	H3	0.0	27.4	3.5	0.016
X5	H5	T4	H3	0.0	23.3	18.0	0.600
G3	H2	T4	H4	0.0	50.8	21.6	0.182
T4	HA3	T4	H4	0.0	19.9	12.0	0.367
X5	H3	T4	H4	0.0	14.3	2.1	0.028
X5	H4	T4	H4	0.0	13.8	3.5	0.066
G3	H2	T4	HA3	0.0	91.8	24.5	0.073
G3	H3	T4	HA3	0.0	54.8	18.9	0.122
G3	H4	T4	HA3	0.0	49.1	4.3	0.008
G3	H5	T4	HA3	0.0	50.5	8.1	0.026
T4	H11	T4	HA3	0.0	53.2	5.1	0.009
T4	H12	T4	HA3	0.0	120.0	6.5	0.003
T4	H2	T4	HA3	0.0	105.0	37.9	0.129
T4	H4	T4	HA3	0.0	52.0	12.0	0.054
T4	H5	T4	HA3	0.0	55.1	5.9	0.012
T4	H61	T4	HA3	0.0	50.4	1.9	0.001
T4	H62	T4	HA3	0.0	57.1	1.8	0.001
X5	H2	T4	HA3	0.0	237.0	6.0	0.001
X5	H5	T4	HA3	0.0	178.0	5.6	0.001
G3	H2	X5	H2	0.0	10.3	9.7	0.891
G3	H3	X5	H2	0.0	26.6	10.5	0.159
G3	H4	X5	H2	0.0	11.5	2.3	0.040
G3	H5	X5	H2	0.0	8.88	12.1	1.870
T4	H61	X5	H2	0.0	10.4	6.4	0.388
T4	H62	X5	H2	0.0	11.9	5.2	0.199
T4	HA3	X5	H2	0.0	14.6	6.0	0.170
G3	H1	X5	H3	0.0	12.0	1.6	0.017
G3	H2	X5	H3	0.0	10.2	4.1	0.175
G3	H3	X5	H3	0.0	12.1	1.1	0.010
G3	H4	X5	H3	0.0	13.3	0.6	0.002
G3	H5	X5	H3	0.0	13.3	0.4	0.001
T4	H11	X5	H3	0.0	12.8	6.1	0.227
T4	H12	X5	H3	0.0	11.9	6.3	0.280
T4	H2	X5	H3	0.0	14.4	6.1	0.177
T4	H3	X5	H3	0.0	13.0	2.4	0.033
T4	H4	X5	H3	0.0	12.1	2.1	0.029
T4	H5	X5	H3	0.0	13.3	0.6	0.002
T4	H61	X5	H3	0.0	13.3	0.2	0.000
T4	H62	X5	H3	0.0	12.1	0.2	0.000
T4	HA3	X5	H3	0.0	14.8	4.3	0.088

G3	H1	X5	H4	0.0	10.2	2.1	0.043
G3	H2	X5	H4	0.0	11.1	1.7	0.025
G3	H3	X5	H4	0.0	15.8	1.1	0.005
G3	H4	X5	H4	0.0	14.0	0.3	0.000
G3	H5	X5	H4	0.0	14.0	0.6	0.002
T4	H11	X5	H4	0.0	15.8	15.1	0.920
T4	H12	X5	H4	0.0	14.1	14.2	1.040
T4	H2	X5	H4	0.0	12.9	8.9	0.479
T4	H3	X5	H4	0.0	11.0	3.5	0.100
T4	H4	X5	H4	0.0	15.8	3.5	0.050
T4	H5	X5	H4	0.0	14.0	0.7	0.003
T4	H61	X5	H4	0.0	14.0	0.4	0.001
T4	H62	X5	H4	0.0	15.8	0.4	0.001
T4	HA3	X5	H4	0.0	15.0	3.2	0.050
G3	H1	X5	H5	0.0	14.9	9.6	0.418
G3	H2	X5	H5	0.0	12.3	2.9	0.058
G3	H3	X5	H5	0.0	17.0	3.5	0.044
G3	H4	X5	H5	0.0	17.0	0.6	0.001
G3	H5	X5	H5	0.0	17.0	2.7	0.026
T4	H5	X5	H5	0.0	12.9	3.0	0.054
T4	H61	X5	H5	0.0	12.9	1.7	0.018
T4	H62	X5	H5	0.0	17.0	1.5	0.009
T4	HA3	X5	H5	0.0	13.3	5.6	0.192

[a] The donor and acceptor nuclei are those on the indirect and direct dimensions of the spectrum, respectively. [b] Refer to Figure S6 for nomenclature. [c] Measured height, calculation error applied, predicted heights, and calculated mean χ^2, as described in (5)). Data measured from a 2D-NOESY recorded with 700ms mixing time on a 3 mM sample at pH 6.0 in 100% (v/v) D_2O and at 5 °C.

Table S10. Summary of χ^2 fits for all structural restraints for residues G3-X5 in HA_4-2AA.

Restraint	Number of restraints	Total χ^2	χ^2/ restraint
$^3J_{HH}$	9	2.8	0.31
NOEs	41	29.6	0.72
noNOEs	94	18.4	0.20
Total	144	50.8	0.35

Table S11. Torsion angle conformational preferences for HA_4-2AA in aqueous solution.

Torsion	Torsion definition [a]	Number of modes [b]	Mean angle (μ) of mode (°)	Libration angle (σ) of mode (°) [c]	Occupancy (π) [d]
**Based on identity of chemical shifts and coupling constants, the solution dynamic 3D structure parameters for the unmodified HA oligosaccharide portion (residues G1-G3) were taken from) (5):**					
G1 C5 – C6	G1 C4 – G1 C5 – G1 C6 – G1 O61	1	98	15	1.00
G1 – S2 Φ	G1 C2 – G1 C1 – S2 O3 – S2 C3	1	171	23	1.00
G1 – S2 Ψ	G1 C1 – S2 O3 – S2 C3 – S2 C2	1	-122	23	1.00
S2 C2 – NA1	S2 C1 – S2 C2 – S2 NA1 – SA CA2	1	120	24	1.00
S2 C5 – C6	S2 C4 – S2 C5 – S2 C6 – S2 O6	2	180	15	0.53
			60	15	0.47
S2 – G3 Φ	S2 C2 – S2 C1 – G3 O4 – G3 C4	1	177	19	1.00
S2 – G3 Ψ	S2 C1 – G3 O4 – G3 C4 – G3 C5	1	145	19	1.00
G3 C5 – C6	G3 C4 – G3 C5 – G3 C6 – G3 O61	1	98	15	1.00
**Solution dynamic 3D structure parameters for the modified HA oligosaccharide portion (residues G3-X5) determined here:**					
Conformationally independent torsions:					
G3 – T4 Φ	G3 C2 – G3 C1 – T4 O3 – T4 C3	1	159	13	1.00
G3 – T4 Ψ	G3 C1 – T4 O3 – T4 C3 – T4 C2	1	-123	3	1.00
T4 C2 – NA1	T4 C1 – T4 C2 – T4 NA1 – T4 CA2	1	114	22	1.00
T4 C3 – C4	T4 C2 – T4 C3 –T4 C4 – T4 C5	1	-60	19	1.00
T4 C4 – C5	T4 C3 – T4 C4 – T4 C5 – T4 C6	1	180	8	1.00
T4 C5 – C6	T4 C4 – T4 C5 – T4 C6 – T4 O6	3	60	19	0.29
			-60	19	0.13
			180	19	0.58
X5 HN11 – X5 C1	T4 C1 – X5 N11 – X5 C1 – X5 C6	1	180	2	1.00
X5 C6 –C7	X5 C1 – X5 C6 – X5 C61 – X5 O611	1	0	2	1.00
Conformationally co-dependent torsions: [e]					
T4 C2 – C3	T4 NA1 – T4 C2 – T4 C1 – T4 O3	3	60	20	0.35
			-60	20	0.08
			-180	20	0.57
T4 C2 – C1	T4 NA1 – T4 C2 – T4 C1 – X5 HN11	2	-60	5	0.92
			-180	5	0.08
T4 C1 – X5 HN11	T4 C2 – T4 C1 – X5 HN11 – X5 C1	5	-127	4	0.35
			117	4	0.35
			-96	4	0.22
			-147	4	0.06
			85	4	0.02
**Dynamic model parameters not measurable by NMR that were assigned modeled values:** [f]					
T4 Me	T4 NA1 – T4 CA2 – T4 CA3 – T4 HA31	3	60	20	0.33
			-60	20	0.33
			180	20	0.33
T4 HO4	T4 C3 – T4 C4 – T4 O4 – T4 HO4	3	60	20	0.33
			-60	20	0.33
			180	20	0.33
T4 HO5	T4 C6 – T4 C5 – T4 O5 – T4 HO5	1	180	20	1.00
T4 HO6	T4 C5 – T4 C6 – T4 O6 – T4 HO6	3	60	20	0.33
			-60	20	0.33
			180	20	0.33

[a] Refer to Figure S6 for atom nomenclature. [b] Modes (macrostates) are classified as distinct if rotation of the bond between the two conformers passes through a van der Waals maximum. [c] The libration amplitude is the standard deviation of the distribution of dihedral angle values from the mean value that the torsion typically adopts for a given mode. [d] The occupancy of each mode is expressed as a proportion of the total for that torsion. [e] Refer to Table 3 for the co-dependency relationships between these 5 modes. [f] Torsions involving hydroxyls and carboxylate groups were given values based on the dynamic 3D structure used for HA_6^{AN} (6), expectations from mining the Cambridge Structural Database (7) and consideration of their positions within each determined conformer.

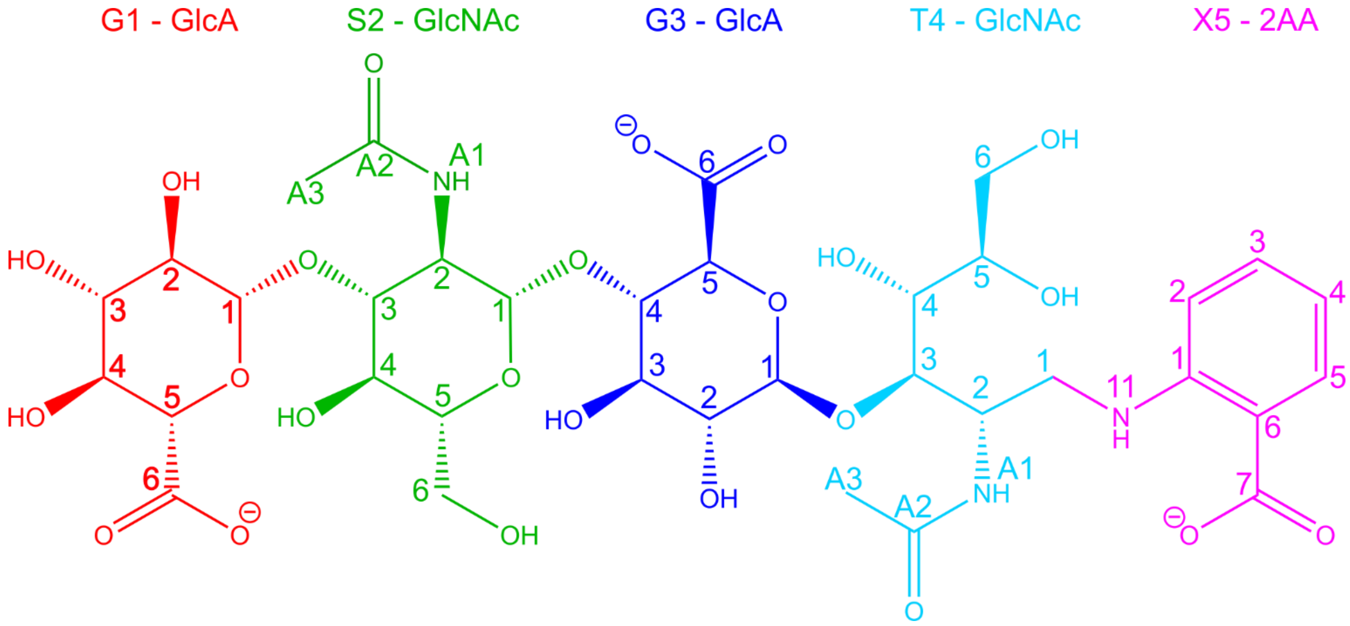

Figure S6. 2D structure and nomenclature of HA$_4$-2AA. The molecule is divided into 5 residues (corresponding to the monosaccharide rings and the 2AA group) and the atom labels within each residue are shown.

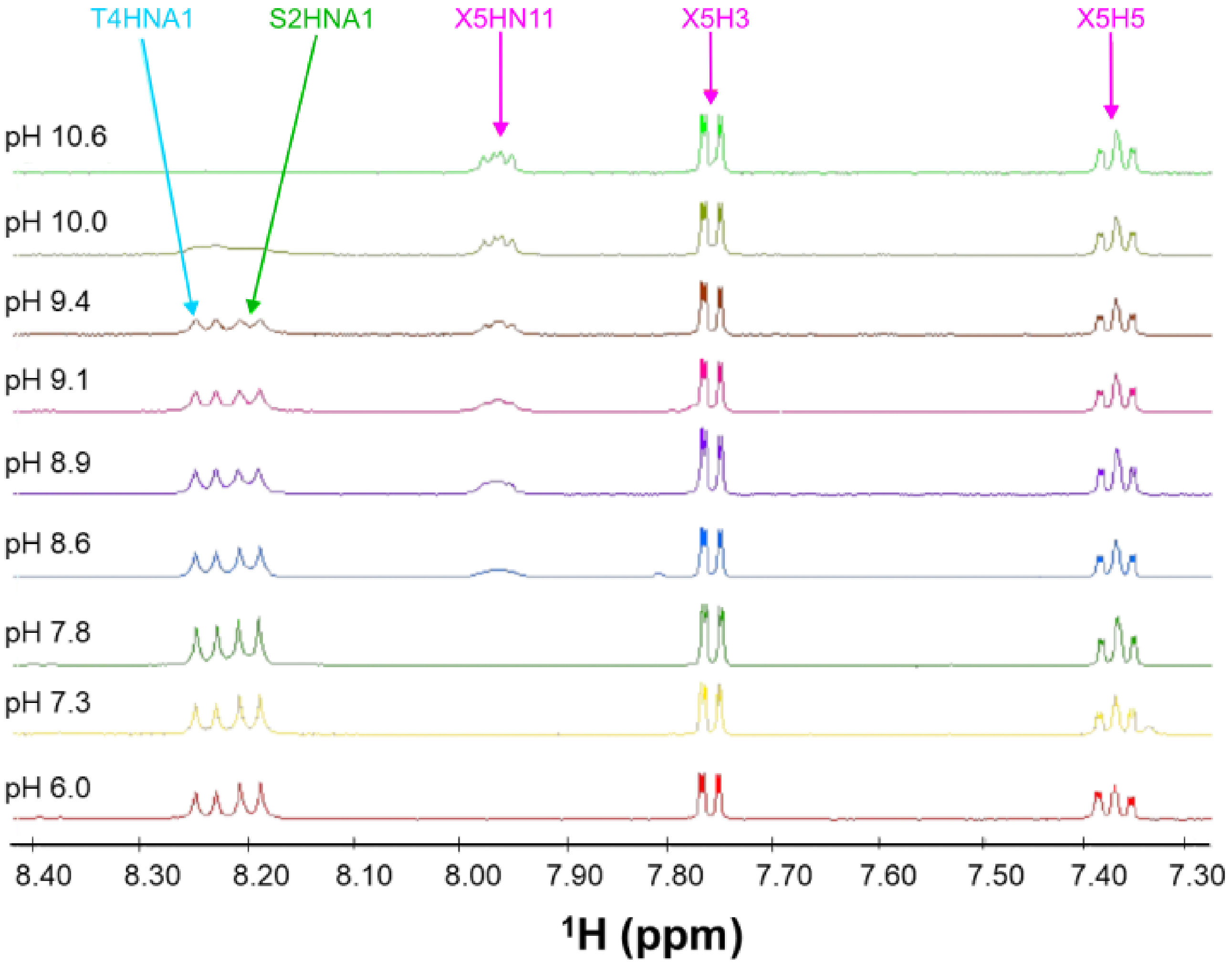

Figure S7. pH titration of HA$_4$-2AA. Portions of 1D ^{1}H spectra recorded at 5 °C on a sample of HA$_4$-2AA (1 mM, 10% (v/v) D_2O) at different pH values (shown from pH 6.0 - 10.6). The difference in exchange rate of the amide (S2 HNA1, T4 HNA1) and aniline (X5 HN11) hydrogens with pH is clearly visible in the differential line-broadening as the pH value is changed. Aromatic hydrogens (X5 H3, X5 H5) do not exchange with solvent and are equally sharp at all pH values.

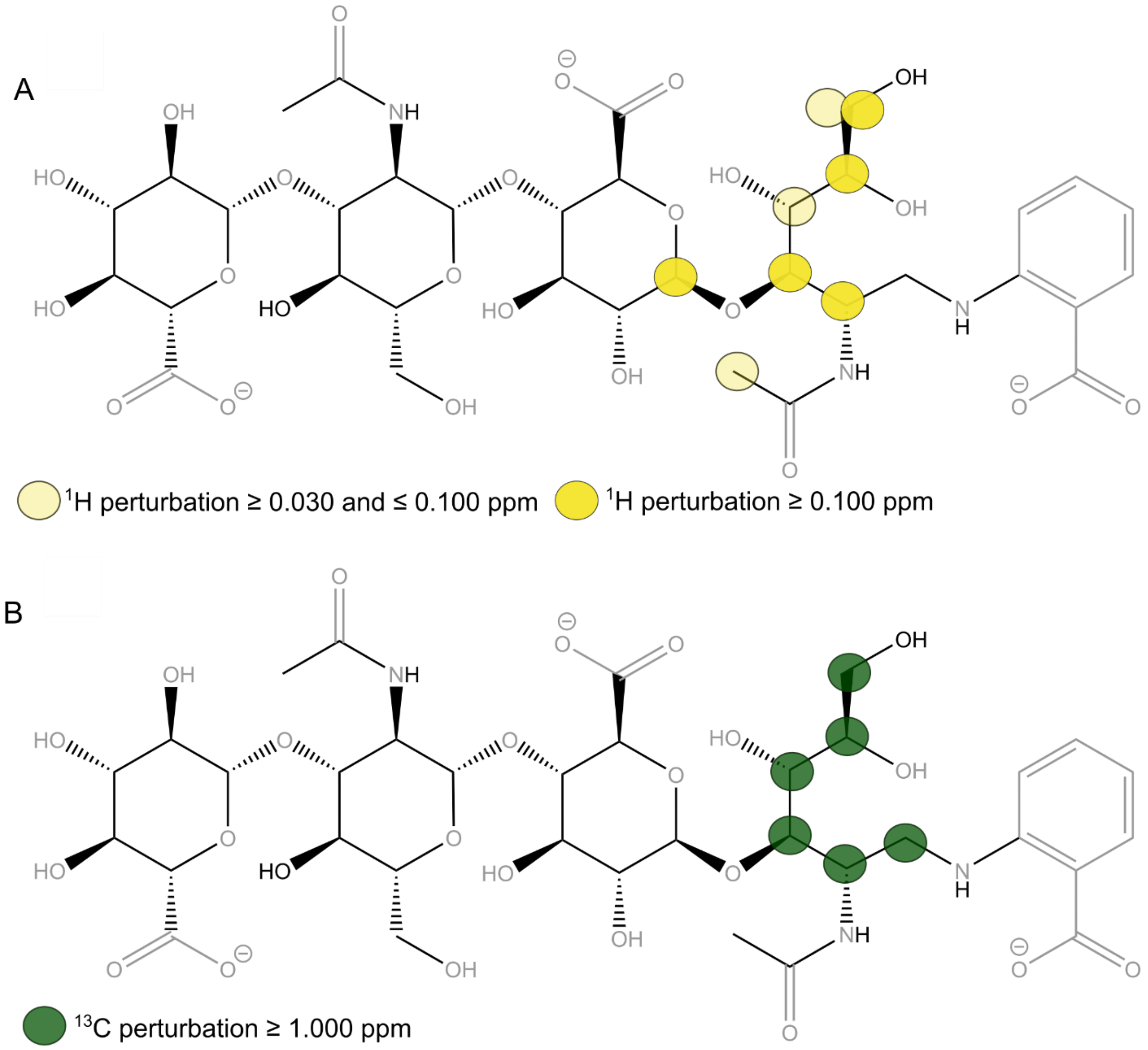

Figure S8. Chemical shift perturbations in HA_4-2AA compared to HA_4^{AN}. The 2D structure of HA_4-2AA is shown with colored circles indicating where in the structure ^{1}H (**A**) and ^{13}C (**B**) chemical shifts are perturbed compared to HA_4^{AN}. Comparison is between HA_4^{AN} at 5 – 20 mM, pH 6.0, 24.4 °C, 10% (v/v) D_2O (values from (3)) and HA_4-2AA at 1 mM, pH 9.4, 5.0 °C, 10% (v/v) D_2O. Atoms in grey are those for which chemical shifts were not measurable (O and H in OH), not measured (N atoms or C in carboxylate groups), or those for which comparison of chemical shift assignment (to HA_4^{AN}) is not possible (*e.g.,* residue X5-2AA).

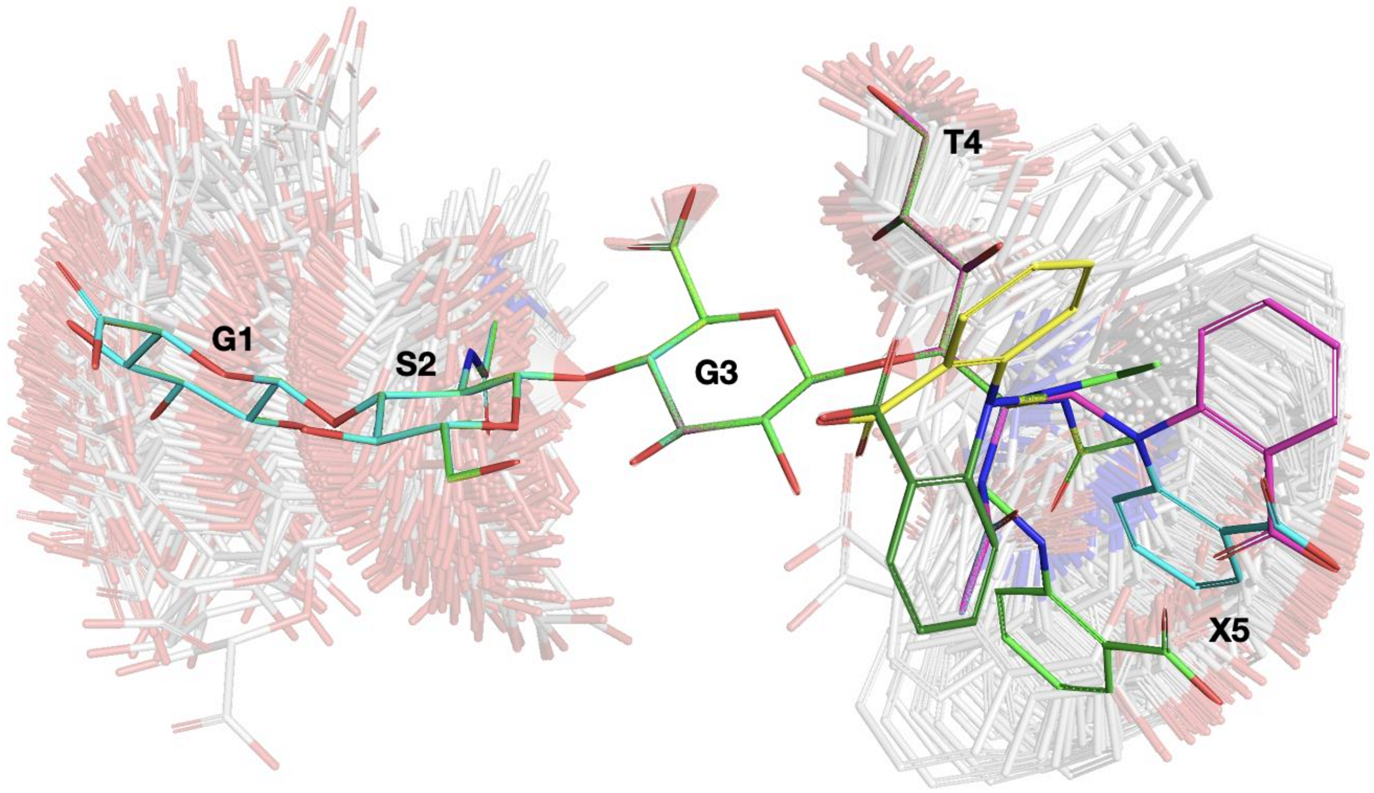

Figure S9. Solution dynamic 3D structure of HA_4-2AA in a conformational ensemble representation. The 5 Conformers generated by the co-dependent behavior of T4 C2 – C3, T4 C2 – C1 and T4 C1 – X5 HN11 (bright conformations) are overlaid together on an ensemble of conformations representing the Gaussian libration about those mean positions (faded conformations). Carbon atoms are colored differently for each Conformer: 1 (green), 2 (cyan), 3 (magenta), 4 (yellow), and 5 (dark green). All Conformers and conformations are overlaid onto the ring atoms of residue G3 (GlcA). Oxygen atoms are shown in red and nitrogen atoms in blue; hydrogen atoms are omitted for clarity. See Figure S8 for residue nomenclature.

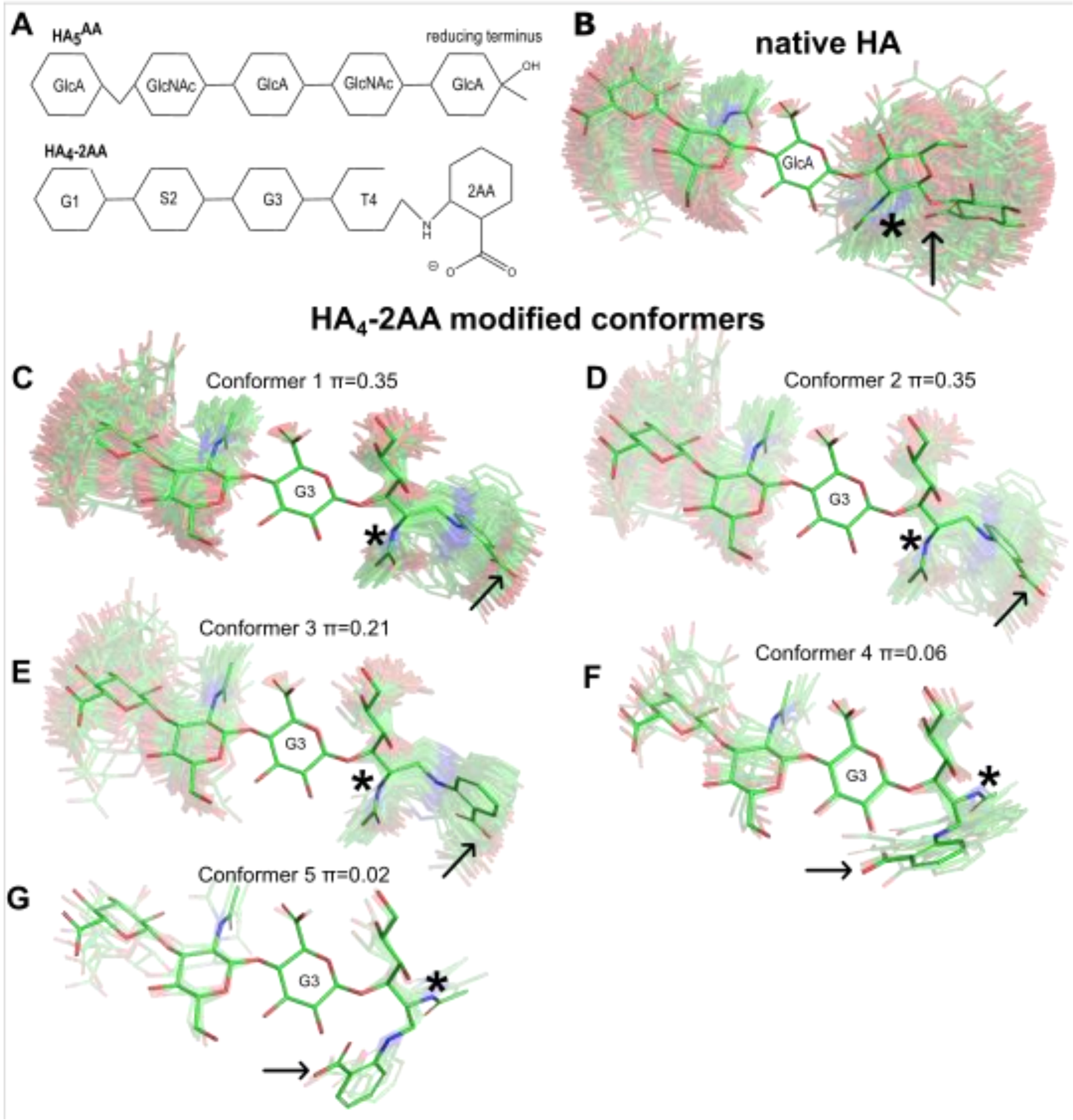

Figure S10. Comparison of solution conformational behavior of HA_4-2AA with HA_5^{AA}. A) 2D structures of HA_5^{AA} and HA_4-2AA, showing residue nomenclature. **B**) Unmodified HA_5^{AA} adopts one conformer in solution (bright conformation) about which it liberates (faded conformations); 250 conformations are shown, determined as described in (55). **C-F)** An ensemble of 250 conformations were determined for HA_4^{AN}-2AA where these adopt 5 different conformers (Conformer 1-5) of differing occupancies (π). Conformers 1-5 are each shown as the mean conformation (bright) along with their associated librational range (faded). All conformations are overlaid on the ring atoms of the central GlcA residue (G3 in HA_4-2AA). Carbon atoms are shown in green, oxygen atoms in red and nitrogen atoms in blue; hydrogen atoms are omitted for clarity.

E. Generation of a model of the HA_6-2AA/Link_TSG6 complex

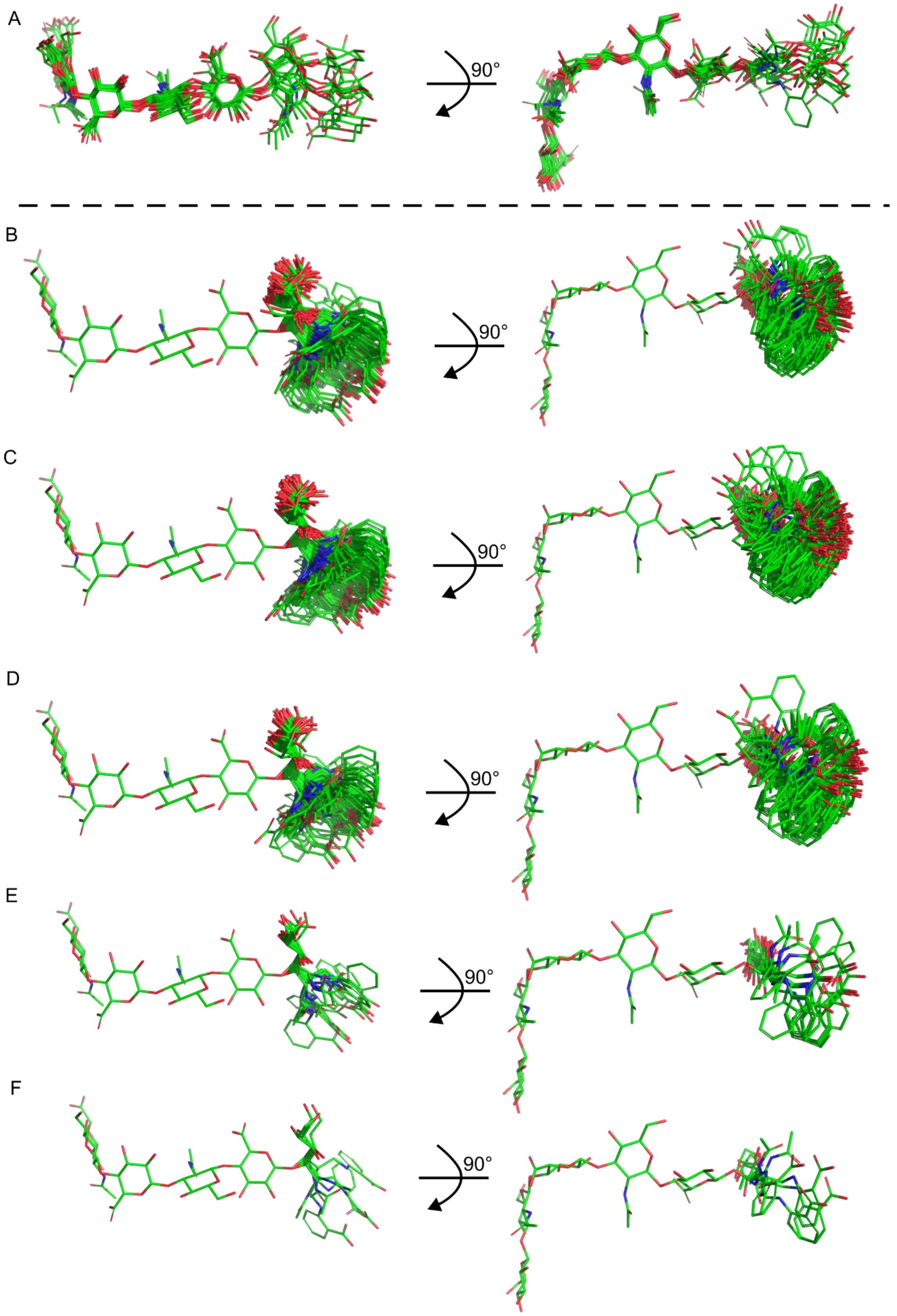

Figure S11. Comparison of conformations of HA_8^{AN} bound to Link_TSG6 with the modeled conformations of HA_6-2AA used for docking into the binding site of Link_TSG6. A) An overlay of the 10 conformations of residues 1-7 of HA_8^{AN} when bound to Link_TSG6 as determined from experiment-based modeling (8). **B-F)**. 250 modeled conformations for the chemically modified residues of HA_6-2AA (6 sugar residues and 2AA) attached to the conserved residues from the lowest energy bound conformation of HA_8^{AN} (residues 1-4); the distribution and number of conformations in B-F represents the behavior and occupancy of each Conformer 1-5 in solution.

F. Supporting Information References

1. Blundell, C. D., Mahoney, D. J., Almond, A., DeAngelis, P. L., Kahmann, J. D., Teriete, P., Pickford, A. R., Campbell, I. D., and Day, A. J. (2003) The Link module from ovulation- and inflammation-associated protein TSG-6 changes conformation on hyaluronan binding. *J. Biol. Chem*. **278**, 49261–49270

2. Teriete, P., Banerji, S., Noble, M., Blundell, C. D., Wright, A. J., Pickford, A. R., Lowe, E., Mahoney, D. J., Tammi, M. I., Kahmann, J. D., Campbell, I. D., Day, A. J., and Jackson, D. G. (2004) Structure of the regulatory hyaluronan binding domain in the inflammatory leukocyte homing receptor CD44. *Mol. Cell* **13**, 483–496

3. Blundell, C. D., Reed, M. A. C., and Almond, A. (2006) Complete assignment of hyaluronan oligosaccharides up to hexasaccharides. *Carbohydr. Res*. **341**, 2803–2815

4. Haasnoot, C. A. G., De Leeuw, F. and Altona, C. (1980) The relationship between proton-proton NMR coupling constants and substituent electronegativities – 1. An empirical generalization of the Karplus equation. *Tetrahedron* **36**, 2783-2792

5. Blundell, C. D., Packer, M. J., and Almond, A. (2013) Quantification of free ligand conformational preferences by NMR and their relationship to the bioactive conformation. *Bioorg. Med. Chem*. **21**, 4976–4987

6. Blundell, C. D., and Almond, A. (2010) Method for determining three-dimensional structures of dynamic molecules. US Patent number US10482997B2

7. Groom, C. R., Bruno, I. J., Lightfoot, M. P., and Ward, S. C. (2016) The Cambridge structural database. *Acta Crystallogr B Struct Sci Cryst Eng Mater*. **72**, 171–179

8. Higman, V. A., Briggs, D. C., Mahoney, D. J., Blundell, C. D., Sattelle, B. M., Dyer, D. P., Green, D. E., DeAngelis, P. L., Almond, A., Milner, C. M., and Day, A. J. (2014) A Refined Model for the TSG-6 Link Module in Complex with Hyaluronan. *J. Biol. Chem*. **289**, 5619–5634
